# Insights into Hsp90 mechanism and *in vivo* functions learned from studies in the yeast, *Saccharomyces cerevisiae*

**DOI:** 10.3389/fmolb.2024.1325590

**Published:** 2024-02-08

**Authors:** Erick I. Rios, Isabel L. Hunsberger, Jill L. Johnson

**Affiliations:** Department of Biological Sciences and Center for Reproductive Biology, University of Idaho, Moscow, ID, United States

**Keywords:** molecular chaperone, Hsp90, cochaperone, *Saccharomyces cerevisiae*, client proteins

## Abstract

The molecular chaperone Hsp90 (Heat shock protein, 90 kDa) is an abundant and essential cytosolic protein required for the stability and/or folding of hundreds of client proteins. Hsp90, along with helper cochaperone proteins, assists client protein folding in an ATP-dependent pathway. The laboratory of Susan Lindquist, in collaboration with other researchers, was the first to establish the yeast *Saccharomyces cerevisiae* as a model organism to study the functional interaction between Hsp90 and clients. Important insights from studies in her lab were that Hsp90 is essential, and that Hsp90 functions and cochaperone interactions are highly conserved between yeast and mammalian cells. Here, we describe key mechanistic insights into the Hsp90 folding cycle that were obtained using the yeast system. We highlight the early contributions of the laboratory of Susan Lindquist and extend our analysis into the broader use of the yeast system to analyze the understanding of the conformational cycle of Hsp90 and the impact of altered Hsp90 function on the proteome.

## Characterization of the two isoforms of Hsp90

Most eukaryotic organisms have two isoforms of cytosolic Hsp90 (Chen et al., 2006). In yeast, the isoforms are Hsc82 and Hsp82, which share 97% amino acid identity. Researchers in the Lindquist lab were the first to clone Hsc82 and analyze the effects of deletion of one or both isoforms (Borkovich et al., 1989). At standard growth temperatures (25°C or 30°C), Hsc82 is one of the most abundant soluble proteins in the cell, but the level of Hsp82 is very low. At elevated temperatures (37°C), the abundance of Hsp82 increases to levels similar to that of Hsc82. We now know that transcription of both *HSC82* and *HSP82* are regulated by transcription factor Heat Shock Factor 1 (Hsf1) (Solis et al., 2016). Deletion of either *HSC82* or *HSP82* resulted in mild growth defects at elevated temperature, but deletion of both resulted in a lethal phenotype. The effect of limiting the abundance of Hsc82/Hsp82 provides some clues about function. In a manuscript from the Lindquist lab (Borkovich et al., 1989), the authors predicted that high levels of expression of Hsc82/Hsp82 help buffer effects of client misfolding due to temperature fluctuations. Subsequent studies supported this hypothesis, showing that reducing the level of Hsc82 or Hsp82 expression to 1%–5% of the wild-type protein levels was sufficient for growth at optimal temperatures, but not elevated temperatures (Picard et al., 1990; Jiang et al., 2013).

The presence of either Hsc82 or Hsp82 is required for viability of yeast, suggesting that the two isoforms have identical or nearly identical features (Borkovich et al., 1989). A more recent study showed that the two yeast isoforms have some differences in ATPase rates, conformational dynamics, and cochaperone interactions. There were also isoform-specific differences in client interactions under both optimal and stress conditions. The Hsp82 isoform was also more thermally stable than Hsc82 at elevated temperatures, consistent with the stress-induced role of that isoform (Girstmair et al., 2019). Isoform-specific differences have also been identified in mammalian cells, which express cytosolic Hsp90 alpha and beta. These differences include patterns of tissue-specific expression, client specificity, and differing interactions with cochaperones (reviewed in (Maiti and Picard, 2022)). Expression of mammalian Hsp90 isoforms alpha and beta in yeast also results in differences in client activity and sensitivity to Hsp90 inhibitors (Piper et al., 2003; Millson et al., 2007).

## Hsp90 structure and the conformational cycle

**FIGURE 1:**
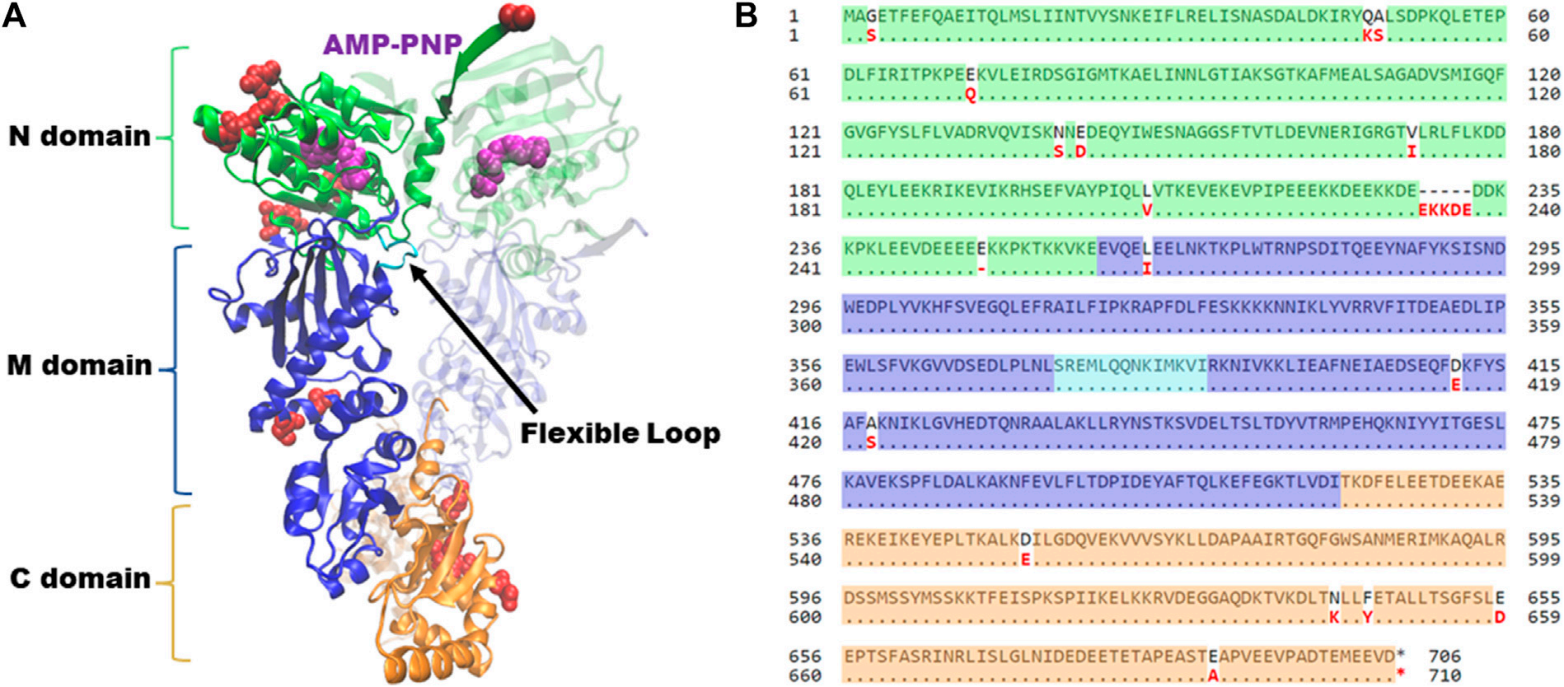
Hsp90 structure and alignment of *Saccharomyces cerevisiae* isoforms. **(A)** Complete structure of *Saccharomyces cerevisiae* Hsc82, in the closed conformation bound to AMP-PNP (magenta) (PDB: 6XLC). Amino-terminal domain (N, green); Middle domain (M, blue); Carboxy-terminal domain (C, orange). Flexible loop in middle domain (loop, cyan). Second translucent monomer in the same color scheme. Residues that differ between Hsc82 and Hsp82 isoforms are noted in red. Figure generated with VMD (Humphrey et al., 1996; Meyer et al., 2003; Liu et al., 2020). **(B)** Alignment of amino acid sequences of *Saccharomyces cerevisiae* Hsc82 (P15108) and Hsp82 (P02829) isoforms utilizing NCBI Protein BLAST tool. Residues have been highlighted corresponding to their respective domains, utilizing the same color scheme as panel 1. Residues that differ between the two isoforms are noted in red.

Hsp90 has three domains; an amino-terminal ATP-binding domain, a middle domain, and a carboxy-terminal domain, which contains the primary site of dimerization (Figure 1A). During the folding cycle, Hsp90 transitions between an open conformation, dimerized only at the carboxy-terminus, and an ATP-induced closed conformation characterized by additional contacts of the amino-terminal domains (Ali et al., 2006). Crystal structures of the amino-terminal domain of Hsp90 showed that the inhibitor geldanamycin and nucleotide bind the same site, and mutational analysis in yeast was used to show that the ability to bind nucleotide is an essential function of Hsp90 (Obermann et al., 1998; Panaretou et al., 1998). The structures of the closed conformation of full-length Hsc82 and Hsp82 are nearly identical (Ali et al., 2006; Liu et al., 2020). As shown in Figure 1B, most of the differences between the two isoforms are located in the amino-terminal domain, which is likely the basis for differences in ATPase activity and sensitivity to Hsp90 inhibitors that bind the nucleotide-binding pocket (Girstmair et al., 2019). Early studies showed that ATP hydrolysis was also essential in yeast, but subsequent studies suggest that nucleotide exchange, rather than hydrolysis, is sufficient for viability (Zierer et al., 2016; Reidy et al., 2023). Additional structural studies, coupled with functional analysis in yeast, identified residues within a flexible loop of the middle domain that play an important role regulating ATP hydrolysis (Meyer et al., 2003). Dimerization of both the carboxy-terminal domains and amino-terminal domains is also critical for function (Wayne and Bolon, 2007; Pullen and Bolon, 2011).

Studies by Nathan and Lindquist identified a panel of amino acid alterations throughout the Hsp82 isoform that confer temperature-sensitive growth when expressed as the only isoform in yeast (Nathan and Lindquist, 1995). Subsequent studies showed that these mutations had diverse effects, either increasing or decreasing ATPase activity, or stabilizing or destabilizing the closed conformation (Prodromou et al., 2000). A variety of studies showed that the rate-limiting step in the Hsp90 cycle is the ability to adopt the closed conformation (Graf et al., 2009; Hessling et al., 2009), and there is evidence for more than one closed conformation (Zierer et al., 2016). Studies in yeast provided evidence that the ability of Hsp90 to progress through various conformations in a timely manner is an essential function. The dwell time of Hsp90 in two separate closed conformations is particularly important (Zierer et al., 2016). Subsequent studies have used large-scale mutational analyses to identify all residues in yeast Hsp90 critical for function (Mishra et al., 2016; Flynn et al., 2020; Cote-Hammarlof et al., 2021). Although differences between the two isoforms have not been studied extensively, studies suggest that select alterations of homologous amino acids in either Hsc82 or Hsp82 have similar effects *in vivo* (Johnson et al., 2007; Kravats et al., 2018; Mercier et al., 2023).

## Hsp90 interaction with cochaperones

Early analysis of Hsp90 complexes identified additional proteins, now called cochaperones, in complex with Hsp90 and client proteins (Smith et al., 1990; Grammatikakis et al., 1999). Studies in the Lindquist lab showed that Hsp90 forms complexes with cochaperones in yeast cell extracts analogous to Hsp90 complexes found in vertebrate extracts (Chang and Lindquist, 1994). Many of the first assays of cochaperone function were conducted in yeast by or in collaboration with members of the Lindquist lab (Kimura et al., 1995; Duina et al., 1996; Chang et al., 1997; Duina et al., 1998). The Lindquist lab was also the first to identify the cochaperones Hch1 and Cns1 (Nathan et al., 1999). Hch1 shares homology with Aha1, the strongest activator of Hsp90 ATPase activity (Panaretou et al., 2002). Structural and functional analysis of individual cochaperones uncovered distinct roles in regulation of Hsp90 ATPase activity and/or Hsp90 conformational changes (reviewed in (Prodromou, 2016; Schopf et al., 2017)). Cochaperones are diverse in terms of the types of domains they contain (Dean and Johnson, 2021). The two most common domains are CHORD-containing proteins and Sgt1 (CS) domains (Garcia-Ranea et al., 2002), as found in p23/Sba1, and tetratricopeptide repeat (TPR) domains (D'Andrea and Regan, 2003), as found in Hop/Sti1 and FKBP51/52. The list of cochaperones continues to grow, with up to approximately 50 cochaperones identified in mammalian cells and approximately 14 in yeast (Sahasrabudhe et al., 2017; Schopf et al., 2017; Backe et al., 2023a). Mutations in some human cochaperones have been linked to a variety of disorders (Dean and Johnson, 2021; Johnson, 2021). Results from the Lindquist lab and others suggest that some cochaperones have preference for clients with certain types of structural domains, such as the preference of Cdc37 for kinase domains (Stuttmann et al., 2008; Taipale et al., 2012; Taipale et al., 2014; Verba and Agard, 2017; Schopf et al., 2019). Some cochaperones also have the ability to act as molecular chaperones and suppress aggregation of proteins such as citrate synthase (Bose et al., 1996; Freeman et al., 1996; Mayr et al., 2000). Further studies are needed to establish the client range of individual cochaperones.

**FIGURE 2:**
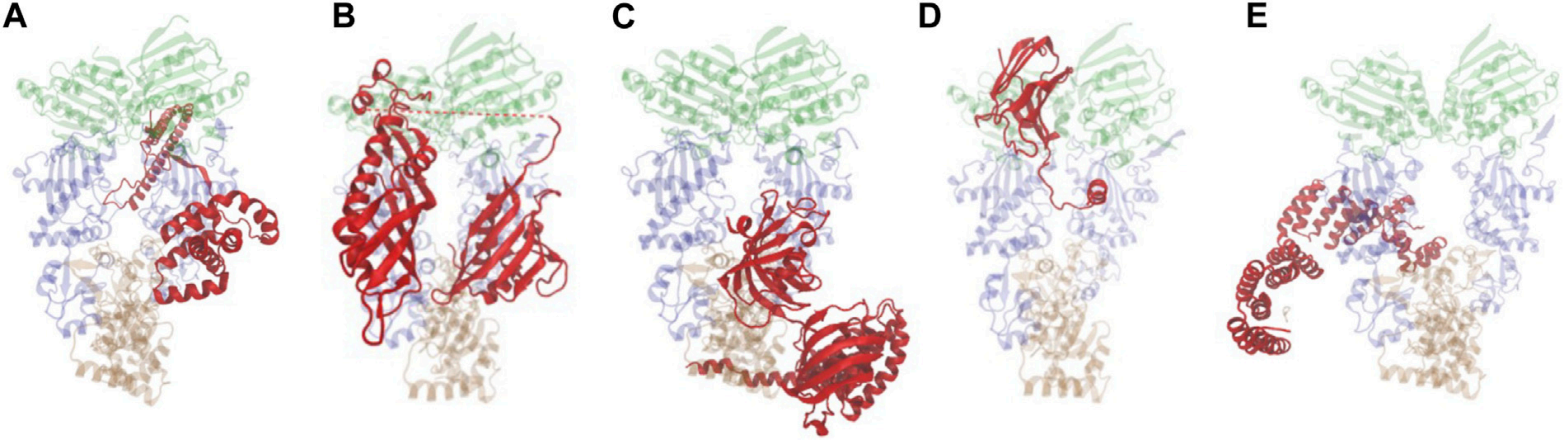
Hsp90-cochaperone structures. Hsp90 is presented as translucent and the domain color scheme is the same as in Figure 1. Cochaperones shown in red. Figures were generated with VMD (Humphrey et al., 1996). **(A)** Cdc37 bound to Hsp90β (PDB: 5FWL (Verba et al., 2016)). **(B)** Aha1 bound to Hsc82 (PDB: 6XLF (Liu et al., 2020)). **(C)**. FKBP51 bound to Hsp90α (PDB: 7L7I (Lee et al., 2021)). **(D)**. p23/Sba1 bound to Hsp90α (PDB: 7KRJ (Noddings et al., 2022)). **(E)**. Hop bound to Hsp90α (PDB: 7KW7(Wang et al., 2022)).

Cryo-EM structures of Hsp90 bound to individual cochaperones, some also in complex with clients are available (Verba et al., 2016; Liu et al., 2020; Lee et al., 2021; Noddings et al., 2022; Wang et al., 2022; Noddings et al., 2023). Some cochaperones directly contact clients through conserved sequences and alteration of those sequences results in reduced client activity. Direct cochaperone-client interactions are likely the underlying basis for previously observed selective effects of cochaperone mutation on client activity in yeast (Lee et al., 2004; Verba et al., 2016; Sahasrabudhe et al., 2017; Biebl et al., 2020; Liu et al., 2020; Noddings et al., 2022; Wang et al., 2022; Noddings et al., 2023). Figure 2 shows a sampling of the diverse nature of cochaperones and how they interact with Hsp90, sometimes using more than one contact site. A more detailed description of their functions is presented elsewhere (Schopf et al., 2017). In the Cryo-EM structure (Figure 2A), Cdc37 binds the middle domain of Hsp90, but in a prior crystal structure, Cdc37 bound the amino-terminal domain in a manner that would inhibit ATP hydrolysis, suggesting that there may be different binding sites depending on the folding cycle (Roe et al., 2004; Verba et al., 2016). As shown in Figure 2B, Aha1, which stimulates ATP hydrolysis, binds the Hsp90 middle domain but using two different domains that bind opposite protomers (Meyer et al., 2004; Liu et al., 2020). The TPR domain of FKBP51 interacts with the sequences in the carboxy-terminal domain of Hsp90 (Figure 2C) (Lee et al., 2021; Noddings et al., 2023). In contrast, p23/Sba1 binds the amino-terminal domain of Hsp90 (Figure 2D) (Weikl et al., 1999; Ali et al., 2006; Noddings et al., 2022). Multiple lines of evidence demonstrate that Sti1/Hop makes contacts with both the middle and carboxy-terminal domains of Hsp90, as shown in Figure 2E (Richter et al., 2003; Rohl et al., 2015; Wang et al., 2022).

**FIGURE 3:**
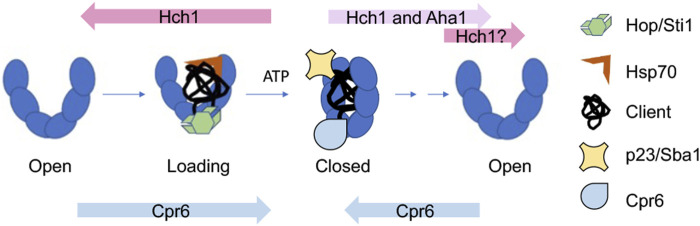
Model of how cochaperones function to regulate and drive progression through the yeast Hsp90 folding cycle. Overexpression or deletion of indicated cochaperones enhance growth defects of yeast Hsp90 mutants defective at progressing through distinct steps in the folding cycle (Mercier et al., 2023). See text for details.

Integration of information about Hsp90 conformational changes and cochaperone function results in a simplified model of how Hsp90 and cochaperones cooperate during the folding of clients, such as the glucocorticoid receptor (GR) (Figure 3). Hop/Sti1, along with Hsp70, targets clients to Hsp90. Hop binds the open conformation of Hsp90 and its release is necessary for progression through the folding cycle (Richter et al., 2003; Wegele et al., 2006; Wang et al., 2022). Aha1 stimulates ATP hydrolysis and promotes structural rearrangements required for adopting the closed conformation (Retzlaff et al., 2010; Li et al., 2013). Other cochaperones such as p23/Sba1 and Cpr6 bind and stabilize the closed conformation (Richter et al., 2004; Johnson et al., 2007). It is likely that some clients have different folding pathways characterized by different targeting cochaperones. For example, protein kinases are targeted to Hsp90 by the cochaperone Cdc37. There are examples of other cochaperones targeting distinct clients, as well as targeting of a single client by different cochaperones (Stepanova et al., 1996; Bansal et al., 2004; Schopf et al., 2019; Biebl et al., 2022; Clerico and Gierasch, 2022).

Over 20 years ago, Nathan and Lindquist described differing impacts of cochaperone overexpression on growth of yeast expressing *hsp82* mutations (Nathan et al., 1999). In particular, overexpression of *HCH1* strongly enhanced or repressed growth defects of different *hsp82* mutants. A recent study that used *hsc82* mutants defective at distinct stages of the folding cycle suggests a simple explanation: there is a phenotypic shift in effects of *HCH1* overexpression or deletion that correlates with whether *hsc82/hsp82* mutants affect steps before or after formation of the closed, ATP-bound conformation (Hohrman et al., 2021; Mercier et al., 2023). Overexpression of *HCH1* enhanced growth defects of mutants that impact formation of the loading complex or closed complex. The hypothesis is that Hch1 destabilizes the closed conformation by reducing Hsp90-nucleotide interaction (Mercier et al., 2019) (Figure 3). However, overexpression of *HCH1*, and in some cases, *AHA1*, rescues growth defects of mutants defective in ATP hydrolysis, likely through aiding conformation changes in Hsp90 and/or nucleotide release (Panaretou et al., 2002; Retzlaff et al., 2010). Other studies examined the effect of Hsp90 mutation on the conformational cycle and the ability of cochaperones, including Aha1, to modulate those effects using purified proteins (Rehn et al., 2016). The role of Hch1 in regulating nucleotide interaction adds additional support for evidence that nucleotide exchange is an essential step in the Hsp90 cycle (Reidy et al., 2023). However, a bigger question is whether mammalian Aha1 has a similar function. Hch1 is only present in some lower eukaryotes (Panaretou et al., 2002), and it has been suggested that a post-transcriptional modification of Hsp90 replaces the role of Hch1 in higher eukaryotes (Zuehlke et al., 2017; LaPointe et al., 2020). Hch1 and Aha1 are not the only cochaperones that have critical roles regulating the folding pathway *in vivo*; deletion of *CPR6* negatively impacts growth of *hsc82* mutants that affect steps early in the cycle, but rescues mutations that appear to be defective in steps that occur after formation of the closed complex. This suggests that Cpr6 promotes formation of the closed conformation, but its release is required for cycle progression (Mercier et al., 2023) (Figure 3). It is unknown whether the mammalian homolog of Cpr6 (Cyp40), or other immunophilins such as FKBP51/52 have a similar role in regulating transition into and out of the closed conformation. However, changes in the relative abundance of Aha1 and immunophilins have been shown to have opposing effects on client fate (Wang et al., 2006; Jinwal et al., 2015; Shelton et al., 2017a; Baker et al., 2017; Shelton et al., 2017b).

One major area of interest today is deciphering the ‘chaperone code’ which seeks to understand how post-translational modifications (PTMs) play a large role in Hsp90 and chaperone function and client fate. PTMs are known to modulate the Hsp90 folding cycle and binding of cochaperones and clients, which may provide further insight for use of small-molecule inhibitors to Hsp90 (reviewed in (Backe et al., 2020)). Studies in *S. cerevisiae* have been indispensable to understanding how PTMs such as phosphorylation, acetylation, SUMOylation, and ubiquitination regulate Hsp90 function. Some the first temperature sensitive mutations of Hsp90 were isolated by the Lindquist lab, T22I and T101I, which occur at residues found to be modified by phosphorylation (Nathan and Lindquist, 1995; Mollapour et al., 2011; Woodford et al., 2016). Both T22 and T101 are conserved threonine residues located within the amino-terminal domain of Hsp90. Phosphorylation of T22 or T101 disrupts binding of the Aha1 cochaperone, leading to decreased ATPase activity, and ultimately affecting client maturation. Interestingly, phosphorylation of T22 decreases binding of Cdc37, while phosphorylation of T101 has been found to increase interaction with Cdc37 (Mollapour et al., 2011; Woodford et al., 2016). Another recently identified temperature-sensitive mutation, S25P, also occurs at a residue in the amino-terminus known to undergo phorphorylation (Mercier et al., 2019; Backe et al., 2023b). SUMOylation and tyrosine phosphorylation of Hsp90 residues have also been shown to affect Hsp90 function (Mollapour et al., 2010; Xu et al., 2012; Beebe et al., 2013; Mollapour et al., 2014). Here we can see that the Hsp90 folding cycle is tightly regulated by PTMs and knowledge of the chaperone code continues to provide insight towards understanding how the dynamic interchange between Hsp90 and cochaperones promotes client maturation.

## Analysis of the functional interaction of Hsp90 with steroid hormone receptors, protein kinases and other clients

Early studies identified Hsp90 in complex with either the glucocorticoid receptor, the progesterone receptor, or the v-src kinase (Bresnick et al., 1989; Kost et al., 1989; Xu and Lindquist, 1993), but at that time, the function of Hsp90 was not understood. The Lindquist lab was part of key studies that showed that yeast Hsp90 is able to chaperone clients from other organisms, indicating Hsp90 function is highly conserved across species. They demonstrated that the activity of the v-src kinase was reduced in yeast lacking *HSC82* (Xu and Lindquist, 1993). In collaborative studies, they used yeast strains engineered to express reduced levels of Hsp90 to demonstrate that Hsp90 was required for the estrogen receptor and GR to activate transcription in response to hormone (Picard et al., 1990). In contrast, they found that mutations in the Hsp70 cochaperone Ydj1 resulted in derepressed GR activity in the absence of hormone (Kimura et al., 1995). This may be due to the role of Hsp70 in unfolding GR prior to refolding by Hsp90 and cochaperones (Kirschke et al., 2014). Amino acid alterations throughout yeast Hsp90 resulted in reduced activity of both GR and v-src, and some mutants also resulted in a sharp decrease of the steady state level of the two clients, supporting a role for Hsp90 in client folding (Nathan and Lindquist, 1995). Once assays for GR receptor and v-src activity became available, they quickly became the standard for monitoring activity of Hsp90 and cochaperone function, elucidating residues critical to ATPase activity, client-activity, and Hsp90-cochaperone interaction (Meyer et al., 2003; Riggs et al., 2003). In general, Hsp90 mutants affect both clients similarly, although some client-specific differences have been observed (Bohen and Yamamoto, 1993; Hawle et al., 2006; Mishra et al., 2016). Hsp90 was also linked to the function of other proteins (Koyasu et al., 1986; Kellermayer and Csermely, 1995; Donze and Picard, 1999; Abbas-Terki et al., 2000), and additional assays of client function in yeast have been developed more recently (reviewed in (Backe et al., 2023a)). Although the list of Hsp90 clients grew, there were doubts about whether it was a general chaperone able to assist any protein, or whether it had more restrictive functions. Studies showed that unlike Hsp70, Hsp90 does not cross-link to native chains emerging from the ribosome (Frydman et al., 1994). To help resolve the question, the Lindquist lab used a unique mutation, *hsp82-G170D*, that becomes rapidly inactivated after a shift to increased temperatures to examine Hsp90 functions. Unlike the effect of more general chaperones, a large increase in cellular aggregates was not detected upon Hsp90 inactivation (Nathan et al., 1997). Moreover, the folding of beta-galactosidase was not significantly affected by Hsp90 inactivation, but Hsp90 was involved in refolding of heat-inactivated firefly luciferase, suggesting Hsp90 exhibits client selectivity. Together, these studies helped to establish Hsp90 as a specialized chaperone with selective clientele. Based on these results, Hsp90 was speculated to play a role in keeping clients inactive until they reached the correct cellular location, or until the cellular protein received some activating signal, such as ligand interaction (Xu and Lindquist, 1993).

## Yeast as a model system to study the extent of Hsp90 functions *in vivo*

**FIGURE 4:**
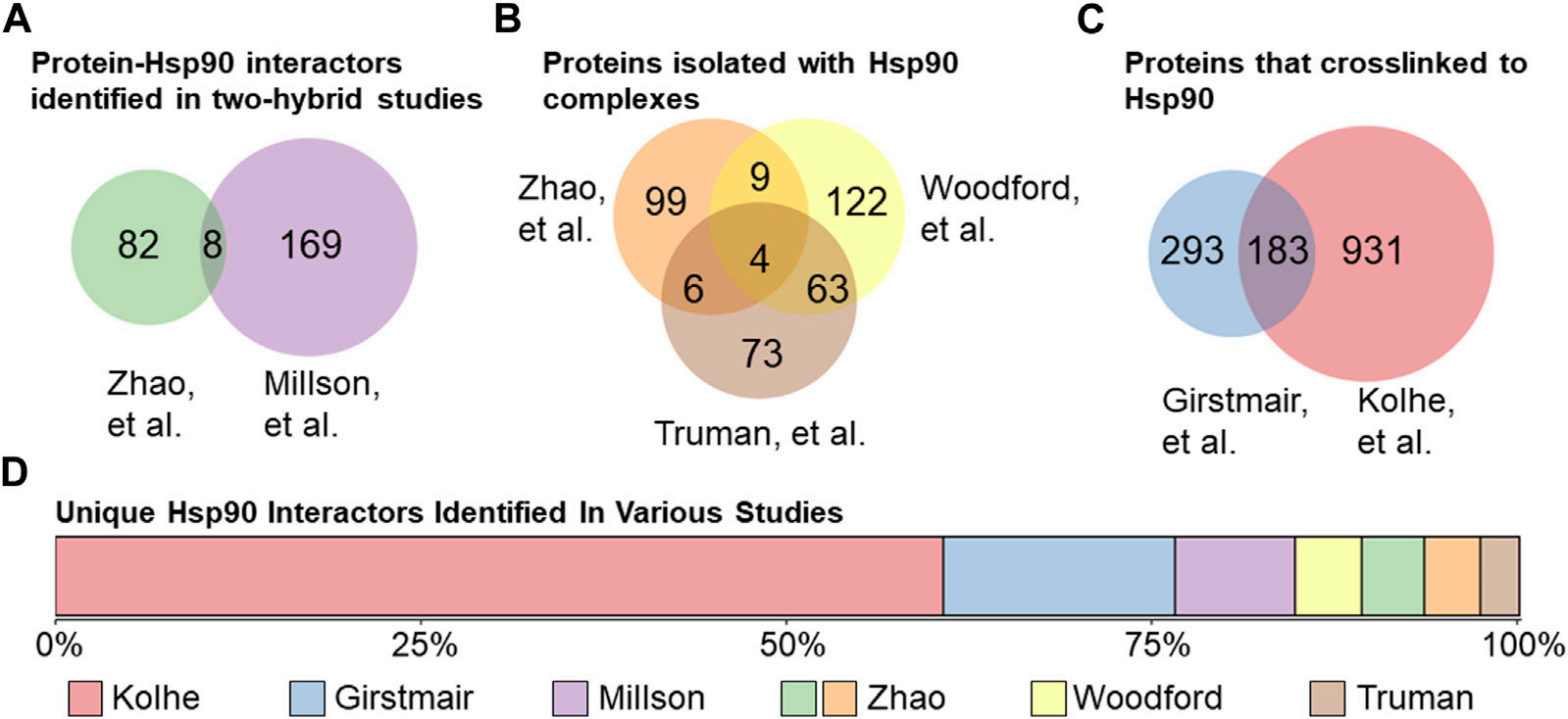
Hsp90 interactors in yeast. **(A)**. Protein physical interactors of Hsp90 identified in yeast two-hybrid studies: Zhao et al. (90 total, left (Zhao et al., 2005)) or Millson et al. (177 total, right (Millson et al., 2005)). 8 protein hits identified in both studies. **(B)**. Proteins isolated in Hsp90 complexes identified in Zhao et al. (118 total, top-left (Zhao et al., 2005)) or Woodford et al. (198 total, top-right (Woodford et al., 2016)) or Truman et al. (146 total, bottom (Truman et al., 2015)). 82 proteins were identified in Hsp90 complexes in at least 2 out of 3 studies. **(C)**. Proteins that have been physically crosslinked to Hsp90 in yeast in Girstmair et al. (476 total, left (Girstmair et al., 2019)) or Kolhe et al. (1114 total, right (Kolhe et al., 2023)). **(D)**. Lack of overlap between high-throughput studies. A total of 1,355 Hsp90 physical interactors were identified only in their respective study, with the majority identified using crosslinking methods.

Several independent studies have sought to interrogate the scope of Hsp90’s interactome, for which the *S. cerevisiae* model organism has proven to be invaluable as it is amenable to high throughput genomic and proteomic approaches. Since Hsp90 interacts with many proteins that are part of signaling cascades (Taipale et al., 2010), it is difficult to distinguish direct client interactions from indirect effects. Two approaches used to identify proteins that directly interact with Hsp90 were yeast two-hybrid studies and isolation of Hsp90 complexes followed by mass spectrometry identification of interactors. For example, one study that used a two-hybrid approach with the E33A point mutation in Hsp90 (Obermann et al., 1998; Panaretou et al., 1998), which abrogates the ATPase activity, to stabilize the normally transient client interactions, found 177 interactions (Millson et al., 2005). That same year, Zhao et al. used full-length or single domains of Hsp90 as bait in a two-hybrid system and observed 90 interactions (Zhao et al., 2005) (Figure 4A). Zhao et al. also used tandem affinity purification to isolate Hsp90 complexes and observed 118 proteins that co-isolated with Hsp90 (Zhao et al., 2005) (Figure 4B). Two studies used metal affinity chromatography to isolate Histidine-tagged Hsp90 complexes. Truman et al. identified 146 Hsp90 partners that interact before and/or after DNA damage (Truman et al., 2015). Similarly, Woodford et al. observed 198 interactors, some of which were dependent on the phosphorylation state of the T101 residue (Woodford et al., 2016). More recent studies used different crosslinking methods to identify proteins that physically interact with Hsp90. As summarized in Figure 4C, one study identified 476 interactors (Girstmair et al., 2019), some of which interacted only at elevated temperatures, while another study identified 1,114 interactors (Kolhe et al., 2023). Collectively, these studies identified 1783 out of the 6,486 (over 25%) yeast proteins as Hsp90 interactors. One of the surprises is the lack of overlap between studies designed to identify direct interactions. Of the 1783, 1,355 were identified in only their respective study (Figure 4D). Over 75% of the unique hits were identified in the crosslinking studies, demonstrating the effective stabilization of transient interactors. In some cases, the lack of overlap may be due to different growth conditions. For example, one of the crosslinking studies harvested yeast in the exponential growth phase (Kolhe et al., 2023), while the other harvested cells in stationary phase (Girstmair et al., 2019). There are extensive transcriptional changes between yeast growth phases (de la Torre-Ruiz et al., 2015; Soontorngun, 2017), and Hsp90 cochaperones have been shown to exhibit modified functions depending on growth phase (Abbas-Terki et al., 2001).

**FIGURE 5:**
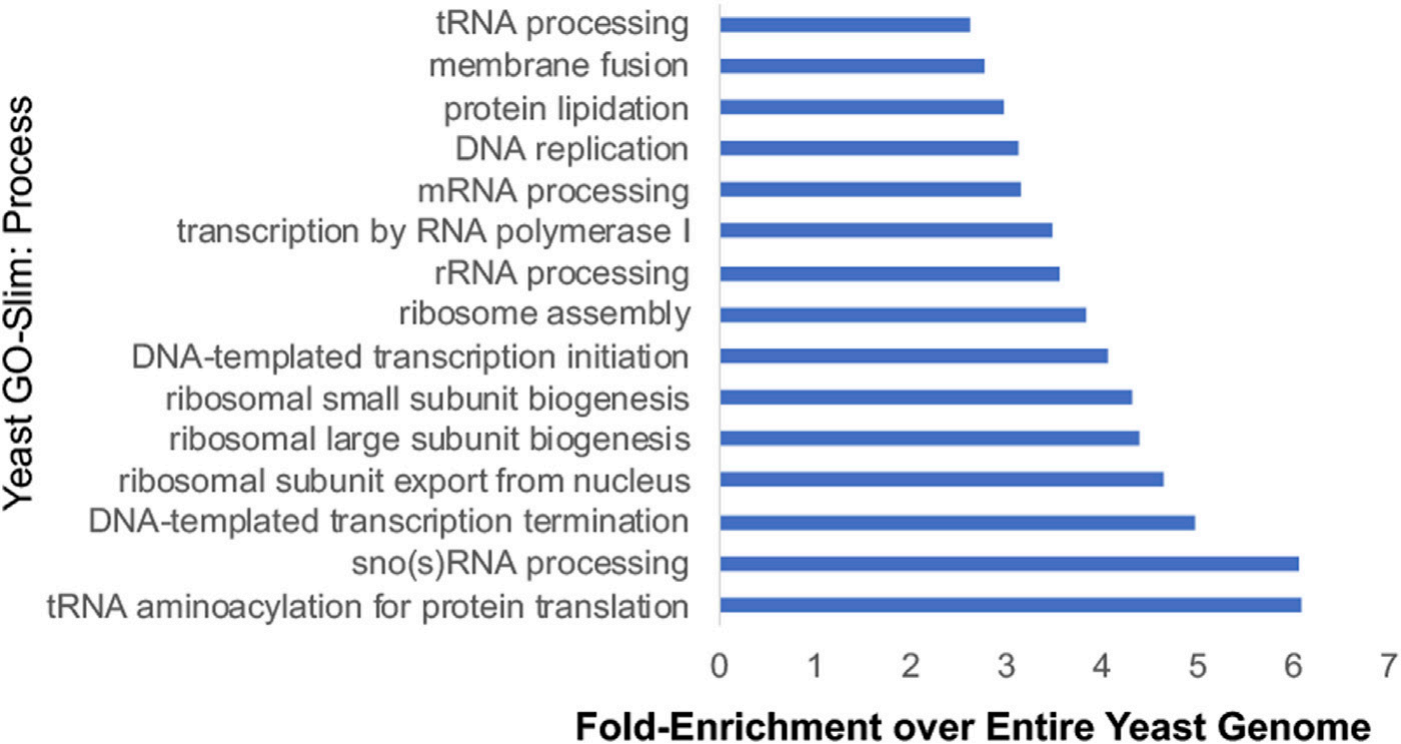
Yeast-Go-Slim analysis of Hsp90 interactors that are encoded by essential genes. Proteins were placed into categories using Yeast-GoSlim Process terms (www.yeastgenome.org), and categories with the highest fold-enrichment over the entire yeast genome are shown. (Giaever et al., 2002; Franzosa et al., 2011; Gopinath et al., 2014; Girstmair et al., 2019; Kolhe et al., 2023).

Additional studies used Hsp90 inhibitors that bind the ATP-binding pocket, or yeast strains expressing either the *hsp82-G170D* temperature sensitive allele or very low levels of Hsp90 to identify the scope of impact of reduced Hsp90 function (Zhao et al., 2005; McClellan et al., 2007; Franzosa et al., 2011; Gopinath et al., 2014). Hsp90 is presumably essential because it has clients that are essential. Systematic analysis of yeast genes identified about 1,100 essential genes (Giaever et al., 2002). Combining the number of proteins encoded by essential genes that physically interact with Hsp90 (Millson et al., 2005; Truman et al., 2015; Woodford et al., 2016; Girstmair et al., 2019; Kolhe et al., 2023), with other identified interactors of Hsp90 (Franzosa et al., 2011), this suggests that Hsp90 interacts with 599 proteins encoded by essential genes. This large percentage (599/1,100, ∼54%) is consistent with a prior study that suggests that Hsp90 clients are enriched for proteins with essential functions (Gopinath et al., 2014). Figure 5 displays the results of Gene Ontology analysis of Hsp90 interactors encoded by essential genes, showing the 15 categories with highest fold-enrichment across the entire yeast genome. Although not shown in this list, some of these proteins are Hsp90 cochaperones or other proteins that have roles in protein folding. This includes the essential cochaperones Sgt1, Cdc37, and Cns1 which have roles in kinetochore function, spindle pole body duplication, and translation, respectively (Schutz et al., 1997; Marsh et al., 1998; Abbas-Terki et al., 2000; Bansal et al., 2004; Davies and Kaplan, 2010; Schopf et al., 2019). Hsp90 interacts with multiple proteins involved in rRNA processing and ribosomal biogenesis and function (Franzosa et al., 2011; Tenge et al., 2014; Kolhe et al., 2023). Hsp90 also has many roles in the nucleus, including transcription by RNA polymerase II, chromatin remodeling and organization, and DNA repair (Flom et al., 2005; Gribun et al., 2008; Zhao et al., 2008; DeZwaan et al., 2009; Truman et al., 2015; Echtenkamp et al., 2016; Gvozdenov et al., 2019; Wang et al., 2020; Omkar et al., 2022). Hsp90 also has demonstrated roles in the cell cycle and the secretory pathway (McClellan et al., 2007). Overall, these studies show the vast roles of Hsp90 at the hub of protein homeostasis (Taipale et al., 2010).

## Conclusion

In summary, work from Dr. Susan Lindquist’s lab was pivotal in establishing *S. cerevisiae* as a model organism to study Hsp90 function. A timeline of some of the contributions from her group, alongside other important works that have led to a deeper understanding of the Hsp90 chaperone system, are shown in Table 1. The yeast system is ideal for testing the effect of Hsp90 or cochaperone mutation on client function. Recent Hsp90 crosslinking studies identified a wide range of potential clients. Additional work is needed to validate those clients and establish whether Hsp90 has a conserved role for chaperoning those clients in yeast and mammalian cells. An intriguing potential is the utilization of the yeast system to study how genomic mutations in Hsp90 clients which result in human disease impact Hsp90 and cochaperone interaction. A yeast model system was previously used to test the functional link between glaucoma-associated mutations in the client WDR36 and the Hop/Sti1 cochaperone (Footz et al., 2009; Footz et al., 2011). Mutations in multiple human Hsp90 cochaperones are associated with disease (Johnson, 2021), some of which affect domains required for Hsp90 interaction (Morgan et al., 2012). Future work is needed to determine whether mutations in the human homologs of proteins that crosslinked to Hsp90 are also linked to human disease. Yeast is also an excellent model system to study the functions of other chaperones, such as Hsp70 and Hsp40 (Kampinga and Craig, 2010; Kampinga et al., 2019), including PTMs of Hsp70 (Nitika et al., 2020).

**TABLE 1:** A timeline of significant discoveries about Hsp90 function.

Year	Significance	References
1989	Hsp90 shown to be essential in yeast	Borkovich et al. (1989)
1990	Reduced levels of Hsp90 compromise steroid hormone receptor activity in yeast	Picard et al. (1990)
1993	Hsp90 governs v-src activity in yeast	Xu and Lindquist (1993)
1994	Hsp90-Hsp70-cochaperone complexes conserved in yeast and vertebrates	Smith et al. (1990), Chang and Lindquist (1994)
1995	Identification of Hsp90 mutations that cause temperature sensitive growth and client defects	Nathan and Lindquist (1995)
1998	Identification of an essential nucleotide binding site in Hsp90 amino-terminal domain	Obermann et al. (1998), Panaretou et al. (1998)
2005	Evidence that Hsp90 interacts with at least 10% of yeast proteome	Zhao et al. (2005)
2006	Full-length crystal structure of closed Hsp90 complex	Ali et al. (2006)
2010–2012	Post-translational modifications, such as phosphorylation, alter cochaperone interactions and client binding	Mollapour et al. (2010), Mollapour et al. (2011), Xu et al. (2012)
2016	Cryo-EM structure of Hsp90-Cdc37-Cdk4 kinase complex	Verba et al. (2016)
2022–2023	Cryo-EM structures of Hsp90-cochaperone-glucocorticoid receptor complexes	Noddings et al. (2022), Wang et al. (2022), Noddings et al. (2023)

Although not discussed here, work from the Lindquist lab was critical in demonstrating a role of Hsp90 in two other important areas relevant to human health. First, members of the Lindquist lab demonstrated a role for Hsp90 in fungal pathogenesis (Cowen and Lindquist, 2005; Cowen et al., 2009). Secondly, they helped to identify the critical roles of Hsp90 in promoting cancerous growth (Whitesell and Lindquist, 2005). Efforts to develop Hsp90 inhibitors as tools to treat cancer are ongoing. An ATP-binding pocket inhibitor was recently approved for use (Kurokawa et al., 2022), and development of Hsp90 isoform specific inhibitors are underway (Khandelwal et al., 2018; Mak et al., 2019; Mishra et al., 2021). A greater understanding of Hsp90 functions that are conserved from yeast to pathogenic fungi to humans will help identify essential functions that may be impacted by Hsp90 inhibition and lead to development of new assays to test for potential negative side effects of those inhibitors.
